# circ0093740 Promotes Tumor Growth and Metastasis by Sponging miR-136/145 and Upregulating DNMT3A in Wilms Tumor

**DOI:** 10.3389/fonc.2021.647352

**Published:** 2021-06-08

**Authors:** Juan Cao, Zhongying Huang, Shunling Ou, Feiqiu Wen, Guocheng Yang, Qiuling Miao, Huang Zhang, Yue Wang, Xiaoxiao He, Yingying Shan, Sixi Liu, Lijuan Jiang

**Affiliations:** ^1^ Shenzhen Children’s Hospital, Shenzhen, China; ^2^ Sun Yat-sen University Cancer Center, State Key Laboratory of Oncology in South China, Collaborative Innovation Center of Cancer Medicine, Guangzhou, China

**Keywords:** circ0093740, DNMT3A, Wilms tumor, circular RNAs, competitive endogenous RNAs

## Abstract

As a research hotspot, circular RNAs (circRNAs) is one type of non-coding RNAs which have many different functions in biological processes. However, there is lack of study investigating the underlying molecular mechanism and the potential roles of circRNAs in Wilms tumor. We conducted a high-throughput microarray sequencing to screen differentially expressed circRNAs in Wilms tumor. A novel circRNA (circ0093740) was identified as a frequently upregulated circRNA in Wilms tumor cells and tissues. Suppression of circ0093740 remarkably inhibited the proliferation and migration ability in Wilms tumor, validated by several experiments. The molecular mechanism of circ0093740 was investigated by luciferase assays and RNA immunoprecipitation assays. The results revealed that circ0093740 promotes the growth and migration ability by sponging miR-136/145 and upregulating DNMT3A. In conclusion, our study discovered the biological role of the circ0093740-miR-136/145-DNMT3A axis in Wilms tumor growth and metastasis which is important for developing new treatment strategy.

## Introduction

Nephroblastoma (also known as Wilms tumor) is a kind of mixed embryonal tumor, which accounts for over 7% of all childhood malignancy and 90% of childhood renal tumors (1). The combination of nephrectomy surgery and chemotherapy was the most common and effective treatment of Wilms tumor. More than 90% of patients with Wilms tumor can be completely cured by systematic therapy (2). However, a part of children with high-risk histology Wilms tumor do not respond to conventional therapy and relapse a few years after surgery (3). Therefore, it is important for researchers to seek for the molecular pathogenesis of Wilms tumor and develop novel treatment strategies to improve outcomes in patients with high-risk Wilms tumors.

CircRNAs are one novel type of single strand non-coding RNAs transcripts in cells, which play very important role in regulating genes *via* different molecular mechanism (4). circRNAs are highly represented in the eukaryotic transcriptome which are ordinated from the back-spliced sequences of exonic or intronic pre-mRNAs (precursor mRNAs) sequences without an upstream head or a downstream tail (5). With a circular structure, they are very stable and resistant to RNA exonuclease which are abundant in most mammalian tissues compared to the linear mRNA transcription (6). In the intracellular microenvironment, circRNAs can regulate the expression of vital oncogenes *via* multiple comprehensive molecular mechanisms, including interacting with proteins, binding microRNAs, and encoding new small molecular proteins (7). Thanks to RNA-seq technology and bioinformatic analysis, lots of circRNAs has been identified (8). circRNAs have been discovered as regulators of a diversity of diseases, including heart failure, neurological disorders, diabetes, and cancers (9). Taking the most famous ciRS-7/cdr1as as an example, this circRNA promotes the growth, migration, chemotherapy resistance, and immune deficiency by sponging miR-7 in multiple types of tumors (10–14). Acts as a tumor suppressor, circFBXW7 is low expressed in tumor tissues which can suppress growth in glioma and breast cancer by sponging miRNA and translating a small 21kda FBXW7 new protein (15, 16). Regulated by TNRC6A, circ0006916 was discovered as tumor promotor in lung cancer cells (17). In addition, circPLK1 were proven as a tumor promoting circRNA by reducing apoptosis in breast cancer (18, 19). The self-renewal and the stemness of colon tumor-initiating cells is enhanced by circCTIC1 by upregulating BPTF-dependent c-Myc expression in colon cancer (20). However, there is lack of study investigating the underlying molecular mechanism and the potential roles of circRNAs in Wilms tumor.

We firstly conducted a high-throughput microarray to screen for the differentially expressed circRNAs in Wilms tumor. We identified a novel circRNA (circ0093740) as a frequently upregulated circRNA in Wilms tumor cells and tissues. Suppression of circ0093740 remarkably inhibited the proliferation and migration ability in Wilms tumor, validated by several experiments. The molecular mechanism of circ0093740 was investigated by luciferase assays and RIP assays. Generally, we discovered the biological functions of the circ0093740-miR-136/145-DNMT3A axis in Wilms tumor growth and metastasis which is important for developing new treatment strategy.

## Materials and Methods

### Clinical Sample Data

Fresh primary Wilms tumor samples and adjacent normal kidney samples were collected from Sun Yat-sen University Cancer Center (SYSUCC) and were frozen in liquid nitrogen at once. This study was approved by the Ethics Committee of the SYSUCC and performed in accordance with the Declaration of Helsinki.

### Cell Culture

Cell lines used in this study including SKNEP1, G401, HANB, and HEK293T were cultured in DMEM (Gibco, USA) containing 10% FBS (Gibco). All cell lines was verified by DNA fingerprinting.

### Western Blot Analysis

The protein was extracted by RIPA and added with PMSF to prevent degradation. The protein was transferred to the PVDF membranes for 2 h at 300 mA and incubated at 4°C with primary antibody (1:1000) overnight and then exposed with the secondary antibody at room temperature for 1 hour. Primary antibody anti-DNMT3A (1:1000, Abcam, USA) and anti-TP53 antibody (1:1000, CST, USA) are used to detect certain protein.

### RT-qPCR Analysis

TRIzo (Invitrogen, USA) was utilized to extract cellular RNA. qRT-PCR assays were conducted with SYBR Premix Ex Taq Kit (Takara, Japan). The primers for circ0093740 are F: 5′- GTGATTGCCGTCCACTCACT-3′; R: 5′- AGTTCTATGGTGGGGTCTGGT-3′. The primers for PCDH15 are F: 5′- AGCACCGGAAGAGTTCTGGAT-3′; R: 5′- ACCACTATTCGCACTTCATGGTA -3′. The primers for ACTB are F: 5′- CATGTACGTTGCTATCCAGGC-3′; R: 5′- CTCCTTAATGTCACGCACGAT-3′.

### Actinomycin D Assay

SKNEP1 Wilms tumor cells were exposed to 5 ug/ml actinomycin D (MCE) to degrade the linear mRNA transcription at 0-, 8-, 16-, and 24-hour time point. Linear PDCH15 mRNA and circRNA circ0093740 were tested by RT-qPCR analysis.

### RNase R Digestion Assay

After 1 ug extracted total RNA of SKNEP1 Wilms tumor cell line was incubated with the RNase R (5 U/ug) or ddH2O for 30 minutes at RT, the remaining RNA solution was purified and quantified by RT-qPCR analysis.

### CCK-8 Assay

SKNEP1 and G401 Wilms tumor cells were digested and then resuspended, and si-circ0093740 (3000 cells per well) and si-control cancer cells (3000 cells per well) were seeded into a 96-well plate. The cells were incubated for two days at 37°C. Afterwards, CCK-8 solution (10 μl) was added to each well of the 96-plate before incubating for two hours.

### Transwell Assay

Overall, 5×10^4^ tumor cells were resuspended and added to the upper chambers (without FBS) and medium (medium containing 20% FBS) was added to the lower chambers. After fix and staining with crystal violet (1.5%), the migrated cells were imaged.

### Luciferase Reporter Assay

SKNEP1 and G401 Wilms tumor cells were seeded into 5 × 10^3^ cells in each well (96-well plate). The predicted miR-136 and miR-145 binding sites of circ0093740, and 3’-UTR of DNMT3A was mutated. Afterwards, the miRNA inhibitors or mimics and constructed reporting vectors (circ0093740-wt/mut or DNMT3A 3’-UTR-wt/mut) were cotransfected into cells for 48 hours before further examination.

### RNA Immunoprecipitation (RIP)

The RIP assays for the AGO2 protein were performed with an anti-Ago2 antibody. The relative expression level of circ0093740, DNMT3A, and miR-136/145 was tested after RNA purification. SKNEP1 and G401 Wilms tumor cells were immediately transfected with MS2bs-circ0093740 vector, MS2bs-circ0093740-mt vector and MS2bs-Rluc vector. The abundance of miR-136/145 was determined after the purification of RNA complexes.

### Mouse Xenograft Assay

SKNEP1 Wilms tumor cells (2×10^7^) were subcutaneously injected into nude mice (four mice for each group, 4-week-old) and treated with intratumoral injection (50 μL si-circ0093740, or si-control) every four days. After 28 days, mice were euthanized. The volume of tumors was estimated every four days according to the following formula: 0.5×width^2^×length. For mouse lung metastasis assay, SKNEP1 cells (3 × 10^5^) were injected through tail veins of nude mice (four mice for each group). The lungs were excised after 8 weeks and the number of metastatic sites were quantified.

### Statistical Analysis

All statistical analysis was performed with SPSS 23.0 software (SPSS, USA). Groups were compared using Student’s t test. Paired t test was used to compare the expression of circ0093740 in two matched groups. All data are reported as the mean ± standard deviation (SD). *P*<0.05 was considered as statistically significant.

## Results

### circ0093740 Is Upregulated in Wilms Tumor Compared to Normal Kidney Tissue

**Figure 1:**
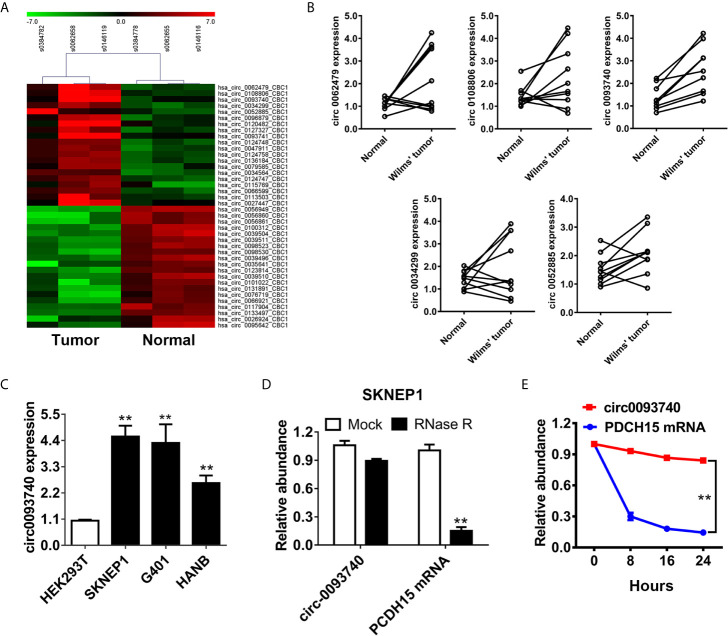
circ0093740 is upregulated in Wilms tumor compared to normal kidney tissue. **(A)** The heat map depicted the differentially expressed circRNAs of three paired Wilms tumor and normal kidney tissue. **(B)** The relative expression level of top five upregulated circRNA between Wilms tumor tissues and normal kidney tissues. **(C)** The relative expression level of circ0093740 in normal HEK293T cell line and Wilms tumor cell lines. **(D)** The circular structure of circ0093740 was tested RNase R assay in SKNEP1 Wilms tumor cell line. **(E)** Circular transcripts of circ0093740 were more stable than its linear PDCH15 mRNA transcripts determined by actinomycin D treated assay in SKNEP1 Wilms tumor cell line. **p < 0.01.

To depict the expression profile of circRNAs in Wilms tumor, a circRNAs microarray assay was conducted using three pairs of Wilms tumor patient tissues and adjacent normal kidney samples (**Figure 1A**). Afterwards, we further examined the expression level of the top five upregulated circRNAs in ten pairs of Wilms tumor patient tissues and normal kidney tissues (**Figure 1B**). Among them, circ0093740 was significantly upregulated in all tumor-normal pairs. circ0093740 was overexpressed in Wilms tumor lines compared to normal kidney cell HEK293T, especially in SKNEP1 and G401 cell lines (**Figure 1C**). We found that the expression of circ0093740 was higher in SKNEP1 and G401, rather than HANB. Therefore, we chose SKNEP1 and G401 for further examination in this study. The actinomycin D assays and the RNase R assays were then carried out to confirm the circular structure and stability of circ0093740. The results revealed that circ0093740 was resisted to RNA exonuclease (**Figure 1D**). circ0093740 has longer half-life span than the linear PDCH15 mRNA in SKNEP1 cell line (**Figure 1E**).

### Suppression of circ0093740 Inhibits the Proliferation of Wilms Tumor Cells

**Figure 2:**
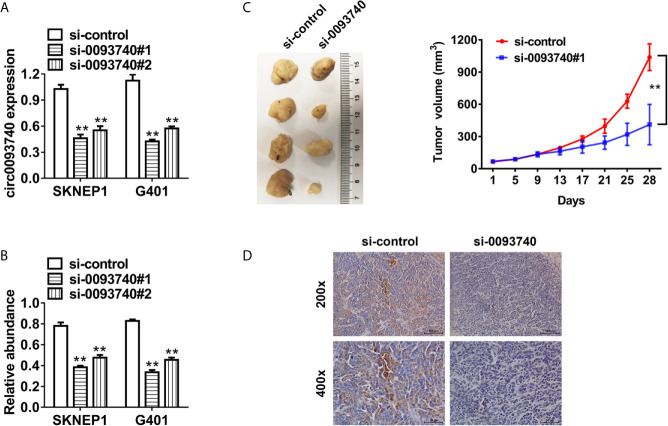
Suppression of circ0093740 inhibits the proliferation of Wilms tumor cells. **(A)** siRNA knockdown of circ0093740 was validated in SKNEP1 and G401 Wilms tumor cell line. **(B)** CCK-8 assays were conducted to evaluate cell proliferation in SKNEP1 Wilms tumor cell line. **(C)** Mouse xenograft models of SKNEP1 Wilms tumor cell line was established. Tumor volume was estimated in every four days. **(D)** The images of Ki-67 IHC expression are presented. **p < 0.01.

We conducted functional assays to explore the potential role of circ0093740 in Wilms tumor progression. circ0093740 was reduced after transfected with siRNAs (sequence anti-back-splicing junction region of circ0093740) which was verified in SKNEP1 and G401 Wilms tumor cell lines (**Figure 2A**). Inhibition of circ0093740 suppressed proliferation ability of SKNEP1 and G401 cell lines *in vitro*, revealed by CCK-8 assays (**Figure 2B**). Further validation of the function of circ0093740 in mouse xenograft assays was conducted. Tumor volumes curves showed that inhibition of circ0093740 could remarkably suppress tumor growth at each time point (**Figure 2C**). Moreover, we found that the Ki67 expression was remarkably decreased in the circ0093740 knockdown group in tumor tissues (**Figure 2D**).

### Suppression of circ0093740 Inhibits the Metastasis of Wilms Tumor Cells

**Figure 3:**
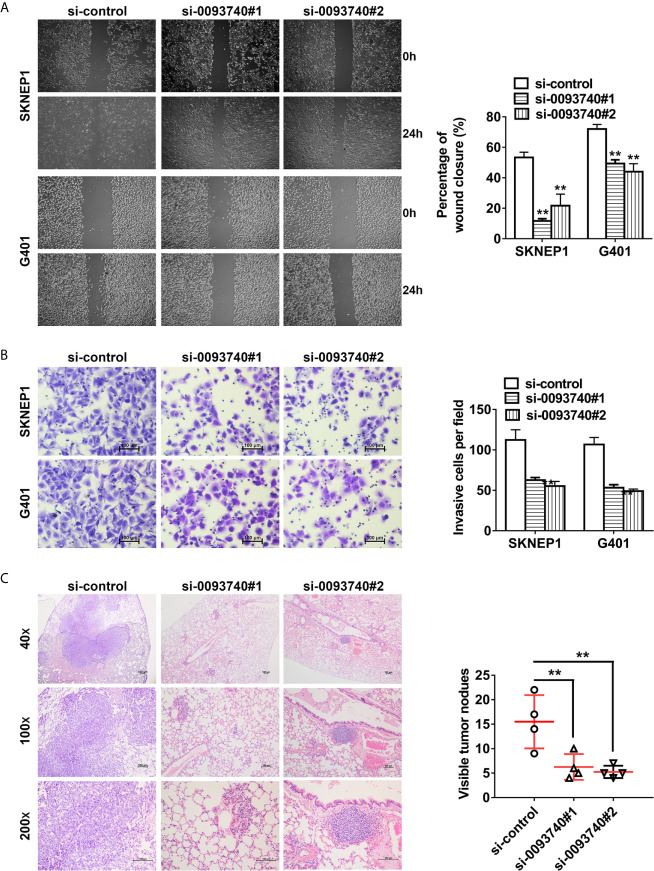
Suppression of circ0093740 inhibits the metastasis of Wilms tumor cells. **(A)** Wound healing assays were conducted in SKNEP1 and G401 Wilms tumor cell line. **(B)** Transwell assay was performed to investigate the migration ability of SKNEP1 and G401 Wilms tumor cell line. **(C)** The number of lung metastases was counted and recorded. HE-stained tumor sections of lung metastases were presented. **p < 0.01.

We next performed migration and invasion associated assays. Downregulation of the expression of circ0093740 could significantly inhibit the percentage of wound healing ability of SKNEP1 and G401 Wilms tumor cells (**Figure 3A**). Transwell assay showed that silence of circ0093740 could reduce the migration ability of SKNEP1 and G401 cells (**Figure 3B**). In consistent with the results of *in vitro* experiments, suppression of circ0093740 could also inhibit the metastatic ability of SKNEP1 and G401 Wilms tumor cells in lung metastasis experiment *in vivo* (**Figure 3C**).

### circ0093740 Serves as a Sponge of miR-136/145 in Wilms Tumor

**Figure 4:**
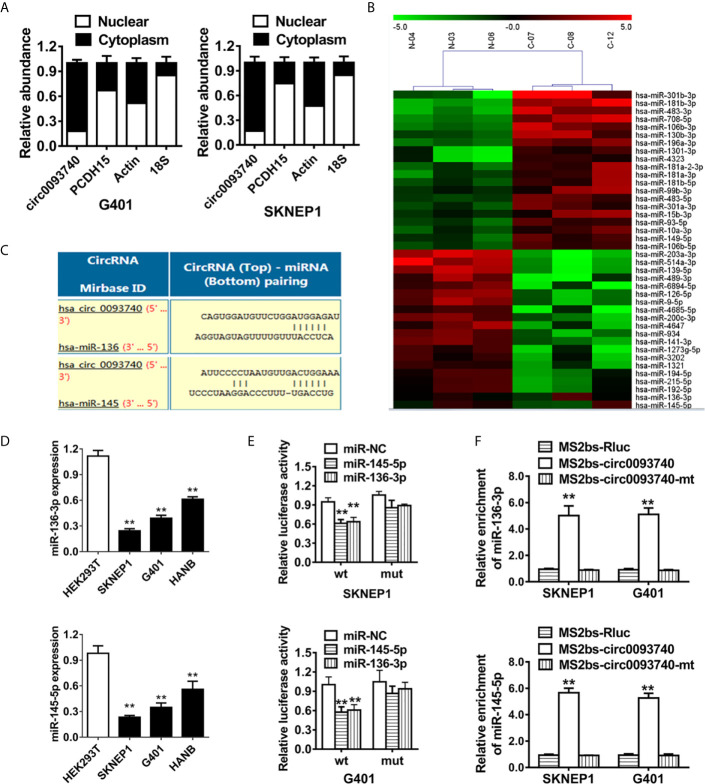
circ0093740 serves as a sponge of miR-136/145 in Wilms tumor. **(A)** 18S, ACTIN, circ0093740 and PCDH15 expression in nuclear and cytoplasmic fractions were analyzed by RT-qPCR. **(B)** miRNA sequencing on three pairs of circ0093740 overexpression and vector samples was conducted in SKNEP1 cell line. **(C)** Predicted binding sites of miR-136/145 within the circ0093740 sequence. **(D)** miR-136 and miR-145 expression in Wilms tumor cell lines compared to HEK293T. **(E)** Luciferase reporter assay was conducted in SKNEP1 and G401 Wilms tumor cell. miR-136 and miR-145 mimics were cotransfected with circ0093740 wild type/mutant luciferase reporter. **(F)** MS2-based RIP assay transfected with MS2bs-circ0093740 vector, MS2bs-circ0093740-mt vector or Rluc control vector. **p < 0.01.

circ0093740 was predominantly expressed in the cytoplasm of cell, detected by qPCR analysis of different cell fraction (**Figure 4A**). As a result, we conducted a miRNA sequencing on three pairs of circ0093740 overexpression and vector samples in SKNEP1 cell line (**Figure 4B**). Circular RNA Interactome database was employed. miR-136 and miR-145 were predicted to interact with circ0093740 and downregulated in circ0093740 overexpression group (**Figure 4C**). In Wilms tumor cell lines, miR-136 and miR-145 were both downregulated detected by RT-qPCR analysis (**Figure 4D**). The results showed that the luciferase activity was significantly reduced after the co-transfection of wild type reporter vectors and miR-136/145 mimics (**Figure 4E**). Ago2-related RIP assays were further conducted to confirm the direct interaction between circ0093740 and miR-136/145. Both miR-136 and miR-145 were predominantly gathered in the MS2bs-circ0093740 vector overexpressed group (**Figure 4F**).

### circ0093740 Facilitates Wilms Tumor Progression Through circ0093740-miR-136/145-DNMT3A Axis

**Figure 5:**
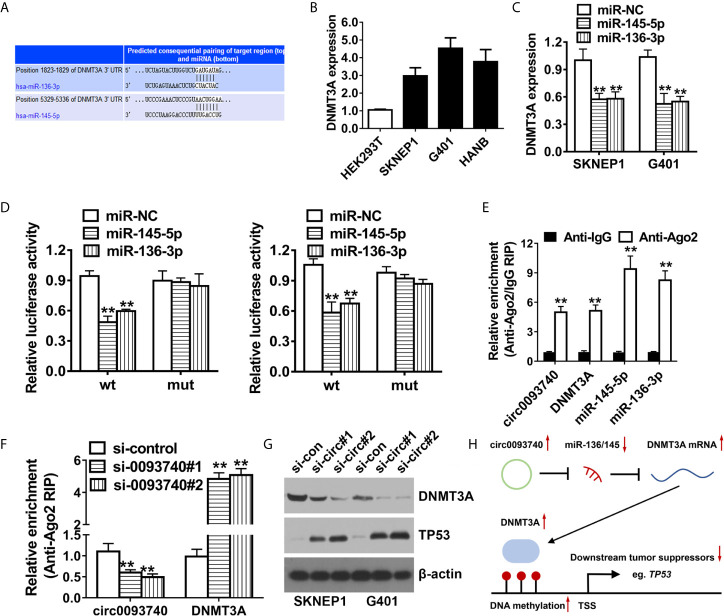
circ0093740 facilitates Wilms tumor progression through circ0093740-miR-136/145-DNMT3A axis. **(A)** According to TargetScan online website, DNMT3A was the putative downstream target of both miR-136 and miR-145. **(B)** DNMT3A expression in Wilms tumor cell lines. **(C)** Expression of DNMT3A transcript was decreased after transfection with miR-136 and miR-145 mimics, detected by qPCR analysis. **(D)** Luciferase reporter assay of SKNEP1 and G401 Wilms tumor cells co-transfected with miR-136/145 mimics and the 3’-UTR of DNMT3A wild type/mutant luciferase reporter. **(E)** Enrichment of circ0093740, DNMT3A and miR-136/145 on AGO2 assessed by RIP assay. **(F)** Enrichment of DNMT3A to AGO2 was increased after silence of circ0093740. **(G)** Knockdown of circ0093740 contributed to the reduction of DNMT3A protein expression in SKNEP1 and G401 Wilms tumor cell. **(H)** The molecular mechanism of Circ0093740 Facilitates Wilms Tumor Progression Through circ0093740-miR-136/145-DNMT3A Axis diagram. **p < 0.01.

Then, TargetScan algorithm was utilized to predict the downstream targeted oncogenes of miR-136 and miR-145. Among the genes, DNMT3A were identified as putative downstream target oncogene of both miR-136 and miR-145 (**Figure 5A**). We conducted qPCR analysis and found that DNMT3A was remarkably upregulated in Wilms tumor (**Figure 5B**). Overexpression of miR-136 and miR-145 contributed to the reduction of DNMT3A mRNA expression level (**Figure 5C**). Luciferase activity was extremely decreased after cotransfection of miR-136/145 mimics and 3’-UTR-DNMT3A reporters in SKNEP1 and G401 Wilms tumor cell lines. After transfection of the mutated reporting vector (mutation of predicted binding site), relative luciferase activity remained unchanged (**Figure 5D**). In addition, we conducted AGO2 related RIP assays which revealed circ0093740, miR-136/145 and DNMT3A were all enriched to AGO2 RNA binding protein in both SKNEP1 and G401 Wilms tumor cell lines (**Figure 5E**). Enrichment to RNA induced silencing complex (RISC) of DNMT3A was remarkably increased after knockdown of circ0093740 (**Figure 5F**). Inhibition of circ0093740 could incredibly decrease the protein level of DNMT3A and increase the protein level of tumor suppressor TP53, analyzing by western blot assays (**Figure 5G**).

## Discussion

With covalently closed loop, circRNAs are widely expressed in mammal tissues with tissue-specific patterns (21). Trans-acting factors and cis-acting elements are essential in the formation of circular structure of circRNAs. Once circRNAs were mainly regarded as to be ‘junks’ generated by incorrected splicing events (22). However, given to the popularity of high-throughput technology, more and more circRNAs are characterized and have proven to play important role in multiple cellular process. In recent years, hundreds of circRNAs were discovered as novel predictive biomarkers and promising treatment targets for cancer therapies (23). circ-CTNNB1 stimulates the Wnt signaling pathway to drive hepatocellular carcinoma progression by encoding a new 370-aa protein. circGNB1 and circRAD18 were identified as oncogenes though the mechanism of ceRNAs in breast cancer (24, 25). Through the HuR-repressed functions of RISC, circular RNA circAGO2 promotes multiple cancers progression (26). Some circRNAs are discovered as tumor suppressors in cancers, though different molecular mechanism (circASS1, circAHNAK1, circITCH, etc.) (27–29). However, there have been no studies investigating the underlying molecular mechanism and the potential biological roles of circRNAs in Wilms tumor up to now.

In this study, we firstly conducted a high-throughput microarray to screen for the circRNA profile in Wilms tumor tissues. circ0093740 was found as a significantly most high expressed circRNA in Wilms tumor cells and tissues. siRNA silencing of circ0093740 remarkably inhibited the proliferation and migration ability of Wilms tumor cells in both *in vitro* and *in vivo* experiments. The results revealed that circ0093740 promotes the Wilms tumor progression by sponging miR-136/145 which contributes to the upregulation of DNMT3A expression (**Figure 5H**).

According to the competing endogenous RNA theory, mRNAs, lncRNAs, and circRNAs can co-regulate with each other (30). miR-136 can suppress cancer stem cell activity to enhance the tumor killing effect of chemotherapy (paclitaxel) by targeting NOTCH signaling pathway in ovarian cancer (31). SMAD3 is downregulated by miR-136 in bladder cancer which is driven by the hypomethylation of PlncRNA-1 promoter (32). In addition, miR-145 is downregulated in colorectal cancer which inhibits the snai1-mediated stemness and sensitizes the cancer cells to radiation (33). miR-145 is regulated by another circRNA CEP128 which can activate MAPK signaling pathway in bladder cancer (34). DNMT3A was upregulated in Wilms tumor which contribute to the epigenetic changes of cancer cells (35). TP53 gene is the most common mutation in Wilms tumor, with an occurrence percentage of 47.5% (36). Hypermethylation of TP53 promoter is driven by DNMT3A which lead to the downregulation of TP53 transcription (37, 38). In our study, DNMT3A was significantly decreased and TP53 was increased after knockdown of circ0093740 in Wilms tumor cells which conforms to the results reported by several published studies.

In conclusion, our study discovered the biological role of the circ0093740 in Wilms tumor growth and metastasis *via* miR-136/145-DNMT3A axis. These findings are very important for developing new treatment strategy and potential prognostic implications in Wilms tumor.

## Data Availability Statement

The original contributions presented in the study are included in the article/supplementary files, further inquiries can be directed to the corresponding authors.

## Ethics Statement

The studies involving human participants were reviewed and approved by the Ethics Committee of the Sun Yat-sen University Cancer Center. Written informed consent to participate in this study was provided by the participants’ legal guardian/next of kin. The animal study was reviewed and approved by Institutional Animal Care and Use Committee of Sun Yat-sen University Cancer Center.

## Author Contributions

All authors listed have made a substantial, direct, and intellectual contribution to the work and approved it for publication.

## Funding

This study was supported by grants from the Clinical Research Project of Shenzhen Municipal Health Commission (SZLY2018015), Guangdong Provincial High-level Clinical Key Specialties (SZGSP012), and Shenzhen Key Medical Discipline Construction Fund (SZXK034).

## Conflict of Interest

The authors declare that the research was conducted in the absence of any commercial or financial relationships that could be construed as a potential conflict of interest.
